# Retraction: Amino acid derived highly luminescent, heteroatom-doped carbon dots for label-free detection of Cd^2+^/Fe^3+^, cell imaging and enhanced antibacterial activity

**DOI:** 10.1039/c8ra90093k

**Published:** 2018-12-03

**Authors:** Andrew Shore

**Affiliations:** Royal Society of Chemistry Thomas Graham House, Science Park, Milton Road Cambridge CB4 0WF UK advances-rsc@rsc.org

## Abstract

Retraction of ‘Amino acid derived highly luminescent, heteroatom-doped carbon dots for label-free detection of Cd^2+^/Fe^3+^, cell imaging and enhanced antibacterial activity’ by Paramita Karfa *et al.*, *RSC Adv.*, 2015, **5**, 58141–58153.

The Royal Society of Chemistry hereby wholly retracts this *RSC Advances* article due to concerns with the reliability of the data in the published article.

A repeating pattern can be observed in the XPS spectra in Fig. 2C in the 390–396 eV range. The XPS data also duplicates data that was presented in another publication, but reported as a different material.^1^

The TEM image in Fig. 3A has been used in another publication, but reported as a different material.^2^

The bacterial growth curves in Fig. 3F illustrate duplication of data, which were reported as different bacterial strains. The growth curves also duplicate data presented in other publications.^3,4^

The fluorescence microscopy images in Fig. 5A and B have been used in another publication, but reported as different materials.^5^

The concentration stability data points in Fig. S2 have been duplicated in Fig. S3 as pH stability data points.

Given the number and significance of the concerns, the validity of the data and, therefore, the conclusions presented in this paper are no longer reliable.

The Royal Society of Chemistry apologises for the fact that these concerns were not identified during the peer review process.

Paramita Karfa, Santanu Patra, Rashmi Madhuri and Prashant K. Sharma oppose the retraction. Ekta Roy, Sunil Kumar and Abhrajyoti Tarafdar were contacted but did not respond.

Signed: Andrew Shore, Executive Editor, *RSC Advances*.

Date: 23rd November 2018.

## Supplementary Material

## References

[cit1] Patra S., Roy E., Madhuri R., Sharma P. K. (2016). ACS Sustainable Chem. Eng..

[cit2] Das T. R., Patra S., Madhuri R., Sharma P. K. (2018). J. Colloid Interface Sci..

[cit3] Roy E., Patra S., Saha S., Madhuri R., Sharma P. K. (2015). RSC Adv..

[cit4] Patra S., Roy E., Karfa P., Kumar S., Madhuri R., Sharma P. K. (2015). ACS Appl. Mater. Interfaces.

[cit5] Roy E., Patra S., Madhuri R., Sharma P. K. (2017). Chem. Eng. J..

